# Coccydynia caused by anatomical variation of the first coccygeal vertebra: a case report

**DOI:** 10.1186/s13256-026-05828-z

**Published:** 2026-01-30

**Authors:** Ping Yang, Xiaoguang Niu

**Affiliations:** 1 Qingdao Medical College, Qingdao University, Qingdao, 266071 Shandong China; 2 Peking University People’s Hospital Qingdao Hospital, Qingdao, 266111 Shandong China; 3 Qingdao Women and Children’s Hospital Affiliated to Qingdao University, Qingdao, 266034 Shandong China

**Keywords:** Coccydynia, Skeletal anomaly, Coccyx excision, Case report

## Abstract

**Background:**

The etiology of pediatric coccydynia is complex. While it is most frequently caused by trauma, a significant proportion of cases are idiopathic, primarily attributed to poor intercoccygeal joint stability and hypermobility of the coccyx. Herein, we report the first case of pediatric coccydynia resulting from a localized developmental anomaly of the coccyx.

**Case presentation:**

A 13-year-old Asian girl was admitted to the hospital owing to 1 year of coccygeal pain during defecation. A computed tomography scan of the sacrococcygeal region revealed a slender protrusion anterior and slightly to the right of the first coccygeal vertebra, extending obliquely in an anteroinferior direction. Pathological findings confirmed it as a normal bony structure. Due to poor response to conservative treatment, the surgeon performed a partial coccygectomy under general anesthesia on the pediatric patient. The procedure involved resection of all bony structures distal to the midportion of the first coccygeal vertebra, including the abnormal protrusion. Intravenous cefuroxime sodium (1 g) was administered within 24 h postoperatively to prevent surgical site infection. The procedure was performed successfully without complications, and the pediatric patient was discharged on the fourth postoperative day. At the 1-month postoperative follow-up, the child reported no significant discomfort.

**Conclusion:**

This case provides new insights into the etiology and pathogenesis of coccydynia.

## Background

Coccydynia refers to pain localized in the coccyx and surrounding tissues, primarily manifested as pain in the coccygeal region while sitting, which may also occur during defecation or sexual intercourse. It is mostly caused by trauma, such as injuries resulting from accidental falls or childbirth, while infections and tumors may also lead to coccydynia [1]. However, one-third of cases are idiopathic, primarily attributed to poor stability of the intercoccygeal joints and excessive mobility of the coccyx [2]. There are very few documented reports on coccydynia in children, and this represents the first reported case of coccydynia caused by localized skeletal abnormalities of the coccyx.

## Case presentation

**Fig. 1:**
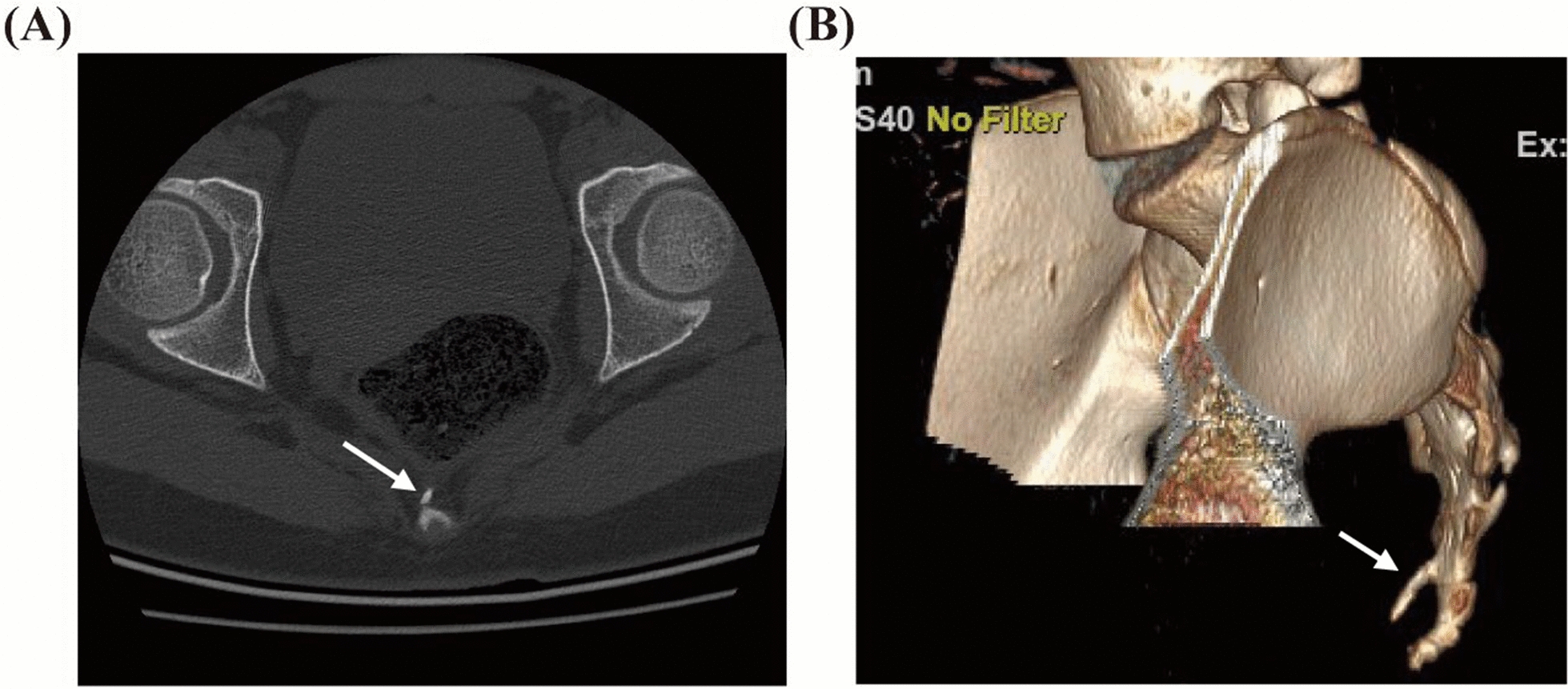
**A** Computed tomography of the sacrum and coccyx with **B** three-dimensional reconstruction; the area indicated by the arrow is the abnormal skeletal structure emanating from the first caudal vertebra

A 13-year-old Asian adolescent female was admitted to the pediatric surgery department in July 2024 for coccygeal pain during defecation that had persisted for 1 year. One year prior, the patient had visited the pediatric surgery outpatient clinic owing to coccygeal pain during defecation. She was continent of urine and stool, with no pain in the lumbosacral region or bilateral lower limbs. The patient had no history of trauma, and there was no family history of similar conditions. Physical examination revealed no obvious tenderness over the sacrococcygeal region, and the surrounding skin appeared normal. Muscle strength and tone in the lower limbs were within normal limits, and no pathological signs were detected; however, during digital rectal examination, an abnormal protrusion was palpated directly posterior to the rectum, accompanied by tenderness. A sacrococcygeal computed tomography (CT) scan showed a slender, obliquely anteroinferiorly oriented protrusion on the anterolateral right side of the first coccygeal vertebral body (Fig. 1). No specific intervention was performed during the initial outpatient visit, and the patient was advised to return for regular follow-up. Over the following year, the patient’s coccygeal pain during defecation worsened, and she occasionally experienced pain in the coccygeal region after prolonged sitting, leading to her admission in July 2024. Upon admission, the patient’s body mass index (BMI) was 20.89 kg/m^2^, and her weight had increased by 5.81 kg over the past year. Notably, the patient maintained favorable psychosocial well-being despite the significant pain symptoms. Given that the patient had been under observation for nearly 1 year with progressively worsening symptoms, a decision was made to proceed with surgical excision.

**Fig. 2:**
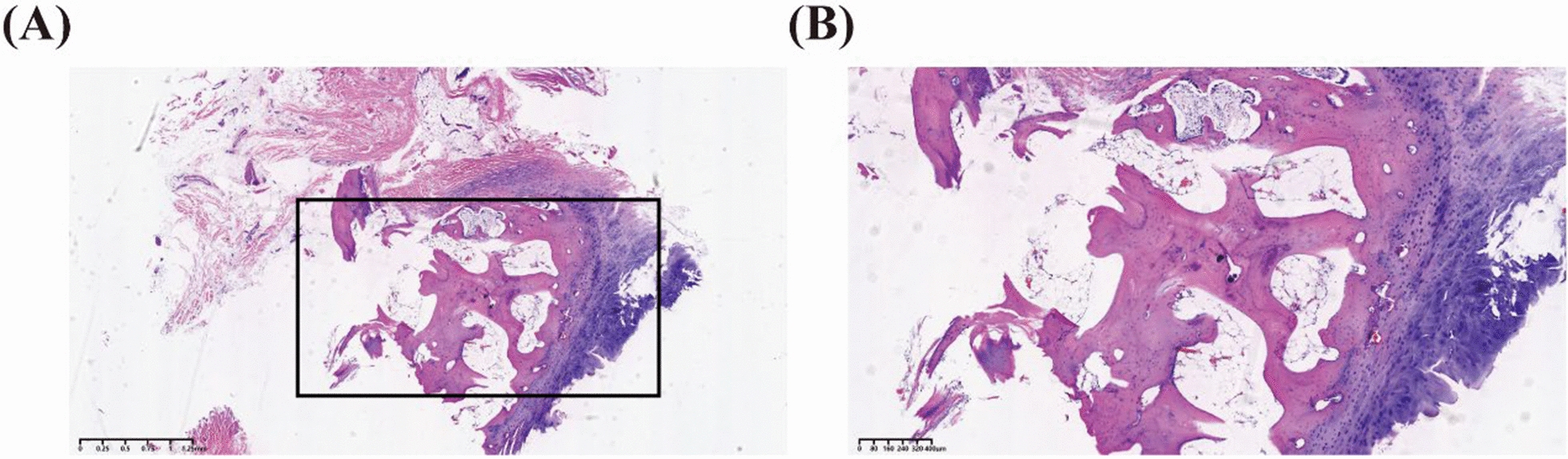
Hematoxylin and eosin staining of the abnormal skeletal structure in the first caudal vertebra. **A** Low-magnification overview. The cartilage tissue is stained blue, while the osseous matrix appears homogeneously eosinophilic (pink). Osteocytes are scattered throughout the field (scale bar, 1.25 mm). **B** Higher-magnification view of the boxed region in (**A**). This image clearly reveals an abundant, interconnected network of trabeculae; bone marrow tissue is evident within the intertrabecular spaces (scale bar, 400 μm)

To avoid nerve injury, it was decided to resect all bony structures distal to the midportion of the first coccygeal vertebra. With the patient in the prone position, a longitudinal incision was made from the distal sacrum to the distal coccyx. Meticulous dissection was performed to prevent nerve damage. Intraoperative findings revealed a bony protrusion approximately 2 cm in length, without cartilage covering its apex. Its base was connected to the first coccygeal vertebra by a disc-like structure, and hypoplasia of the first coccygeal vertebra was also observed. Intravenous cefuroxime sodium (1 g) was administered within 24 h postoperatively. Pathological examination confirmed that the anomalous structure was primarily composed of bone, cartilage, and bone marrow tissue (Fig. 2). Given the absence of trauma and supported by the pathological diagnosis, the etiology of the coccydynia was identified as an osseous abnormality owing to a developmental anomaly in the first coccygeal vertebra. At the 1-month postoperative visit, the patient was asymptomatic for pain and reported satisfaction with the treatment results.

## Discussion and conclusion

Unlike previously reported causes of coccydynia, this case reveals a novel etiology—an abnormal bony protrusion from the first coccygeal vertebra, resembling a coccygeal spur. Anatomical differences in the coccyx between sexes may explain the higher prevalence of coccydynia in females [3]. Woon *et al*. found that the female coccyx is often shorter, straighter, and more prone to posterior dislocation, which can cause pain [4]. In the present case, developmental dysplasia of the first coccygeal vertebra led to posterior dislocation of the second coccygeal vertebra. Furthermore, obesity has been identified as a predisposing factor for coccydynia [5]. The patient’s body weight increased by nearly 6 kg over the past year, which may have contributed to the exacerbation of her pain. The diagnosis of coccydynia is primarily based on patient history and clinical examination, with tenderness potentially elicited during digital rectal examination. In addition, imaging modalities such as sacrococcygeal radiography, CT, and magnetic resonance imaging can aid in confirming the diagnosis [6].

Currently, there is no definitive expert consensus regarding the management of coccydynia, with treatment options ranging from conservative measures and pharmacotherapy to surgical intervention [7]. Coccydynia can be either acute or chronic. In acute cases, over 90% of patients experience spontaneous improvement within several weeks to months with conservative management [8]. Treatment outcomes in adolescents are comparable to those in adults. Kalstad *et al*. advocate for a conservative approach prioritizing the use of seating aids, oral analgesics, and corticosteroid injections, reserving surgical options for cases refractory to these measures [9]. Surgical intervention, involving partial or total coccygectomy, is generally considered a viable option for patients with persistent coccydynia unresponsive to conservative treatment [10]. Multiple studies have confirmed the efficacy of coccygectomy for trauma-induced coccydynia [11, 12]. The most common complication following coccygectomy is surgical site infection. While perioperative antibiotic prophylaxis is used to reduce this risk, no standardized regimen for antibiotic administration has been established [13]. In this case, 1 g cefuroxime sodium was administered intravenously within 24 h postoperatively as prophylactic measure against surgical site infection.

This case reveals that coccygeal skeletal anomalies represent a novel etiology of coccydynia in children. However, the underlying pathogenesis requires further investigation. Given that conservative management provided negligible benefit to this patient, surgical intervention, specifically coccygectomy, should be considered a viable therapeutic option, providing supporting evidence for the efficacy and safety of coccygectomy in pediatric patients.

## Data Availability

Not applicable.

## References

[1] Lee SH, Yang M, Won HS, Kim YD. Coccydynia: anatomic origin and considerations regarding the effectiveness of injections for pain management. Korean J Pain. 2023;36(3):272–80. 37394271 10.3344/kjp.23175 PMC10322656

[2] Sukun A, Cankurtaran T, Agildere M, Weber MA. Imaging findings and treatment in coccydynia - Update of the recent study findings. Rofo Fortschr Geb Rontgenstr Neuen Bildgeb Verfahr. 2024;196(6):560–72. 37944937 10.1055/a-2185-8585

[3] Aggarwal A, Kumar S, Kumar D. Factors influencing the evaluation and management outcomes of coccygodynia: a literature review. J Back Musculoskelet Rehabil. 2013;26(2):105–15. 23640311 10.3233/BMR-2012-00355

[4] Woon JTK, Perumal V, Maigne JY, Stringer MD. CT morphology and morphometry of the normal adult coccyx. Eur Spine J. 2013;22(4):863–70. 23192732 10.1007/s00586-012-2595-2 PMC3631051

[5] Maigne JY, Doursounian L, Chatellier G. Causes and mechanisms of common coccydynia: role of body mass index and coccygeal trauma. Spine. 2000;25(23):3072–9. 11145819 10.1097/00007632-200012010-00015

[6] Daily D, Bridges J, Mo WB, Mo AZ, Massey PA, Zhang AS. Coccydynia: a review of anatomy, causes, diagnosis, and treatment. JBJS Rev. 2024;12(5):e24. 10.2106/JBJS.RVW.24.00007 38709859

[7] Andersen GØ, Milosevic S, Jensen MM, Andersen MØ, Simony A, Rasmussen MM, *et al*. Coccydynia-the efficacy of available treatment options: a systematic review. Global Spine J. 2022;12(7):1611–23. 34927468 10.1177/21925682211065389 PMC9393997

[8] Milosevic S, Andersen GØ, Jensen MM, Rasmussen MM, Carreon L, Andersen MØ, *et al*. The efficacy of coccygectomy in patients with persistent coccydynia. Bone Joint J. 2021;103-B(3):542–6. 33641429 10.1302/0301-620X.103B3.BJJ-2020-1045.R2

[9] Kalstad AM, Knobloch RG, Finsen V. The treatment of coccydynia in adolescents: a case–control study. Bone Jt Open. 2020;1(5):115–20. 33225284 10.1302/2633-1462.15.BJO-2020-0017 PMC7677095

[10] Aarby NS, Trollegaard AM, Hellberg S. Coccygectomy can be a treatment option in chronic coccygodynia. Ugeskr Laeger. 2011;173(7):495–500. 21320414

[11] Pennekamp PH, Kraft CN, Stütz A, Wallny T, Schmitt O, Diedrich O. Coccygectomy for coccygodynia: does pathogenesis matter? J Trauma. 2005;59(6):1414–9. 16394915 10.1097/01.ta.0000195878.50928.3c

[12] Mouhsine E, Garofalo R, Chevalley F, Moretti B, Theumann N, Borens O, *et al*. Posttraumatic coccygeal instability. Spine J. 2006;6(5):544–9. 16934725 10.1016/j.spinee.2005.12.004

[13] Sagoo NS, Haider AS, Palmisciano P, Vannabouathong C, Gonzalez R, Chen AL, *et al*. Coccygectomy for refractory coccygodynia: a systematic review and meta-analysis. Eur Spine J. 2022;31(1):176–89. 34694498 10.1007/s00586-021-07041-6

